# The taxonomic significance of ddRADseq based microsatellite markers in the closely related species of *Heracleum* (Apiaceae)

**DOI:** 10.1371/journal.pone.0232471

**Published:** 2020-05-07

**Authors:** Mehdi Daemi-Saeidabad, Abdolali Shojaeiyan, Adam Vivian-Smith, Hans K. Stenøien, Mohsen Falahati-Anbaran

**Affiliations:** 1 Department of Plant Sciences, School of Biology, College of Science, University of Tehran, Tehran, Iran; 2 Department of Horticultural Science, Faculty of Agriculture, Tarbiat Modares University (TMU), Tehran, Iran; 3 Department of Forest Genetics and Biodiversity, Norwegian Institute for Bioeconomy Research, Ås, Norway; 4 NTNU University Museum, Norwegian University of Science and Technology, Trondheim, Norway; National Cheng Kung University, TAIWAN

## Abstract

Many studies on *Heracleum* have shown poor correspondence between observed molecular clusters and established taxonomic classification amongst closely related species. This might reflect both unresolved taxonomy but perhaps also a lack of good genetic markers. This lack of appropriate and cost effective species-specific genetic markers hinders a resolved relationship for the species complex, and this in turn causes profound management challenges for a genus that contains both endemic species, with important ecological roles, and species with an invasive potential. Microsatellites are traditionally considered markers of choice for comprehensive, yet inexpensive, analyses of genetic variation, including examination of population structure, species identity, linkage map construction and cryptic speciation. In this study, we have used double digest restriction site associated DNA sequencing (ddRADseq) to develop microsatellite markers in *Heracleum rechingeri*. Genomic DNA from three individuals were digested with *Sbf1* and *Nde1* and size selected for library construction. The size-selected fragments were sequenced on an Ion Torrent sequencer and a total of 54 microsatellite sequences were bioinformatically confirmed. Twenty five loci were then tested for amplification, resulting in 19 of these being successfully amplified across eight species, comprising both the so-called thick-stemmed species (*H*. *persicum*, *H*. *rechingeri*, *H*. *gorganicum* and *H*. *lasiopetalum*), and thin-stemmed species (*H*. *anisactis*, *H*. *pastinasifolium* and *H*. *transcaucasicum*). Both Bayesian and distance-based clustering, and principal coordinate analyses clearly separated these into two groups. Surprisingly, three *H*. *pastinacifolium* populations were not separated from populations of the morphologically similar endemic species, *H*. *anisactis*, suggesting lack of genetic differentiation. Likewise, high genetic similarity was found between *H*. *persicum* and *H*. *rechingeri* populations, questioning taxonomic separation at the species level between these taxa. Further analyses are needed to re-evaluate the taxonomic significance of observed morphological variability currently applied to distinguish these sister taxa. Nevertheless, our results represent progress in the effort to develop cost-efficient molecular tools for species discrimination in this genus.

## Introduction

In many plant species complexes, factors such as hybridizations, phenotypic plasticity and high variability across the geographical distribution range makes species identification based on morphological characters challenging [1–3]. *Heracleum* is a taxonomically complex genus, and previous investigations have found incongruent results between observed molecular clusters, morphological data and taxonomic classifications [4–6]. Lack of appropriate species-specific genetic markers hinders progress to untangle clear relationships within a species complex in a cost-effective way [2–4, 6]. This is particularly challenging for a genus with species having important ecological roles, or which are rare, or alternatively invasive, or of special interest for conservation management.

The ecological significance of the *Heracleum* in terms of the negative effect on the local biodiversity has recently been reported in its invasive range in northern Europe [7]. Beside its significant impact on ecosystem, various species of the plant are used for variety of purposes which include as a fodder crop, ornamental use, food additives like spices, and in pharmaceutical applications [8, 9]. Yet certain species also present a human health risk mainly due to the production of phototoxic derivatives in the exuded stem and leaf sap. Correspondingly, the eradication of noxious *Heracleum* species in human populated areas is considered resource-demanding [10].

Molecular studies have shown that the seemingly cosmopolitan genus *Heracleum* (Apiaceae) is not monophyletic [5, 11]. There is also extensive variation associated with genetic, chemical and morphological traits at various geographical levels within and among species [3–5, 12]. The genus consists of more than 100 species found mainly in the West-Asian/Caucasus and Syno-Hymalian regions, the former region being suggested as a center of origin for the genus [3, 11, 13]. Eight species, including *Heracleum persicum* Desf. ex Fisch., *H*. *gorganicum* Rech. F., *H*. *rechingeri* Manden., *H*. *anisactis* Boiss. & Hohen., *H*. *pastinacifolium* C. Koch, *H*. *rawianum* C. C. Towns., *H*. *taranscaucasicum* Manden., and *H*. *antasiaticum* Manden have an Iranian distribution range. In addition, *H*. *lasiopetalum*, a morphologically clearly distinct species, occurs in an alpine region of the Zagros mountains in the western parts of Iran.

These eight species mainly grow in mountainous river banks, moist grasslands and in open patches across the Hircanian forest. Among them, *H*. *rechingeri*, *H*. *gorganicum* and *H*. *anisactis* are reported as endemic species to Iran. Taxonomically, the eight species are divided into three sections: Pubescentia (*H*. *persicum*, *H*. *rechingeri* and *H*. *gorganicum*), Villosa (*H*. *antasiaticum*) and Wendia (*H*. *pastinacifolium*, *H*. *anisactis*, *H*. *transcaucasicum* and *H*. *rawianum*) with each showing a significant morphological differentiation between the sections [14]. On the other hand, the species within each section are reported to be morphologically very similar. For instance, the main morphological character differentiating the Pubescentia and Wendia sections is primarily the thickness of the stem. Furthermore, based on diagnostic taxonomic characters described in Mozaffarian et al. [15] Iranian *Heracleum* species can be classified into two major groups, based on the size of the plant and the stem diameter, namely the “thin-stemmed” (*H*. *pastinacifolium*, *H*. *anisactis*, *H*. *transcaucasicum* and *H*. *rawianum*) and the “thick-stemmed” (*H*. *persicum*, *H*. *rechingeri*, *H*. *gorganicum*). However, recent molecular studies have shown lack of distinctiveness among species within these suggested groups [12].

Genetic markers can be used to clarify how diversity is partitioned within the species complex. Microsatellite markers, short segments of DNA consisting of 2 to 6 base pair (bp) repetitive motifs, may be particularly useful. The main features of these markers include co-dominant inheritance, a high mutation rate, and thus often appreciable variability within and between populations observed in a range of Eukaryotes [16–20]. Another important attribute is presumed selective neutrality of microsatellite markers, sometimes even when found within genic regions [21]. Besides high frequency throughout the genome, and often high variability among individuals, their utility also includes a highly consistent amplification within and among closely related species.

Microsatellites have been extensively used to study species complexes and cryptic speciation, particularly in cases with unclear and unresolved phenotypic relationships [e.g. 2, 22]. For instance, species-specific markers have successfully been applied to discriminate between large and small-glanded individuals of the *Dalechampia scandens* species complex (Euphorbiaceae) [23]. Likewise, such markers have been applied to delimitate the species boundaries in genus *Phoenix* (Arecaceae), showing that the *P*. *dactylifera* clade, unexpectedly, is very distinct from the related species of *P*. *atlantica* and *P*. *canariens* [24]. Additionally, it has been shown that microsatellite markers are often readily transferrable between species [2], but this is not always the case [e.g. 22].

In recent years, next generation sequencing (NGS) techniques have been widely adopted to identify molecular markers including both microsatellite and single nucleotide polymorphism (SNP) in non-model organisms. This can be done at low cost and in a short time, even without an associated reference genome [25, 26]. Furthermore, Restriction Associated DNA sequencing (RADseq), a technique for reproducibly subsampling a target genome [27], has made it possible to evaluate population genomic data for virtually any organism to a high degree of accuracy, with longer sequence reads and in a cost-effective nature [27, 28].

In this study, we wanted to use ddRADseq to identify and develop microsatellite markers to help resolve the *Heracleum* species complex. Specifically we wanted to develop microsatellite markers first, based on the *H*. *rechingeri* genome, and subsequently try to utilize these markers in other closely related species of *Heracleum*. We wanted to test whether thick-stemmed species complex including *H*. *rechingeri* and *H*. *persicum* and *H*. *gorganicum* represent a distinct genetic lineage, and also whether we are able to discriminate endemic thin-stemmed species *H*. *anisactis* and *H*. *transcacasicum* from the widespread *H*. *pastinacifolium*.

## Materials and methods

### Plant materials and ddRADseq analysis

First, leaf tissue from three individuals of *Heracleum rechingeri* were collected from a population in Fandoghlu (Namin, Ardebil province, Iran) and immediately placed on silica gel (Table 1). *H*. *rechingeri* has been treated as a separate species with a few distinct morphological traits, including the shape of mericarp and stylopodium compared to the widespread sister taxa *H*. *persicum* (see Table 2 extracted from Flora Iranica). The species occurs in a narrow distribution range in the western parts of Alborz mountains in northern Iran [15].

**Table 1:** Natural populations of *Heracleum* species used in screening polymorphic microsatellite loci.

Species	Region	Locality	Abbreviation	No. samples	No. alleles	Altitude (m a.s.l.)	Longitude(E)	Latitude(N)
*H*. *rechingeri*	Ardabil	Heyran	H.rech_Heyr	5	7	1575	48°34'18.81"	38°24'57.37"
*H*. *rechingeri*	Ardabil	Asalem	H.rech_Aslm	2	6	1155	49°48'7.42"	39°37'20.28"
*H*. *persicum*	Ardabil	Anbaran	H.pers_Anbr	5	8	1711	48°27'44.83"	38°31'27.68"
*H*. *persicum*	Maragheh	Kordehdeh	H.pers_Krdh	3	8	2016	46°25´45.16"	37°30´25.58"
*H*. *persicum*	Maragheh	Gharatloo	H.pers_Ghrt	2	8	1973	46°25´24.92"	37°29´20.90"
*H*. *persicum*	Maragheh	Yayshahri	H.pers_Yshh	3	7	1982	46°20´58.10"	37°34´28.20"
*H*. *anisactis*	Eastern Azerbaijan	Kandovan	H.anis_Kndv	3	10	2350	46°15´45.40"	37°47´03.89"
*H*. *anisactis*	Eastern Azerbaijan	Liqvan	H.anis_Liqv	3	11	2051	46°28´02.37"	37°47´43.79"
*H*. *anisactis*	Damavand Mount	Damavand	H.anis_Dmvd	2	10	3210	52°6´46.33"	35°54´3.92"
*H*. *trasnscaucasicum*	Kalibar	Babak Castle	H.tran_Babk	3	13	2122	46°58´50.65"	38°50´12.87"
*H*. *pastinacifolium*	Ardabil	Gardane Almas	H.past_Aslm	3	12	2144	40°48´0.06”	35°37´1.91”
*H*. *pastinacifolium*	Ardabil	Nir	H.past_Nyr	3	10	1820	53°47´57.66"	59°37´3.27"
*H*. *pastinacifolium*	Mazandaran	Alamkuh	H.past_Alkoh	2	11	2850	51°. 22.22"	25°36´8.32"
*H*. *antasiaticum*	Harazroad	Zangouleh	H.anta_Zngl	3	7	2462	22°51´47.76"	13°36´15.62"
*H*. *lasiopetalum*	Chelgerd	Kohrang	H.lasi_Kohrg	3	6	2750	50°2´57.78"	27°32´4.68"
*H*. *gorganicum*	Golestan	ShirinAbad	H.gorg_Shrn	2	8	1180	55°5´31.47"	36°50´25.43"
*H*. *sphondylium*	Norway	Trondheim	H.spho_Trhn	2	7	40	10°24´40.11"	63°25´36.49"

**Table 2:** Variation in key morphological traits of taxonomic importance amongst the *Heracleum* species studied. The data is reproduced from Flora Iranica [14].

Species	Section	The shape of mericarp	The shape of stylopodium	Length of commissural vittae	Presence (+) and absence (-) of dorsal vittae	The number of umbellate within blossom	The number or the shape of bracteole	Plant height	Stem Thickness
*H*. *persicum*	Pubescentia	Ovoid	Long conical	2/3 mericarp	+	30–50	Numerous	1–1.5m	Thick
*H*. *gorganicum*	Pubescentia	Long-elliptic	Conical	1/3 mericarp	+	20–25	Numerous	1–1.5m	Thick
*H*. *rechingeri*	Pubescentia	Long-ovoid	Conical	1/3 mericarp	+	25–50	Narrowly lanceolate	1.5m	Thick
*H*. *transcaucasicum*	Wendia	Elliptic	Long conical	1/2 mericarp	-	15–18	Lanceolate	30-100cm	Thin
*H*. *pastinacifolium*	Wendia	Ovoid	Conical	1/2 mericarp	-	14–30	Small	70-100cm	Thin
*H*. *anisactis*	Wendia	Elliptic	Long conical	1/3 mericarp	-	6–9	Minute	35-100cm	Thin
*H*. *antasiaticum*	Villosa	Long-elliptic	Long conical	1/3 mericarp	+	15–30	Few	1–1.5m	Thick
*H*. *lasiopetalum*	Lasiopetala	Ovoid	Long conical	1/3 mericarp	+	10–30	Few	30-100cm	Thin

DNA was extracted from the dried tissues using the ENZA SP Plant DNA Kit as specified by the manufacturer’s protocol (Omega Bio-Tek, Norcross, USA) and eluted in water. DNA was quantified using the broad range Qubit assay (Thermofisher Scientific) and normalized to a working concentration of 5 ng ul^-1^. We used the double digest Restriction site Associated DNA (ddRADseq) protocol as described by Vivian-Smith and Sønstebø [29] from which we developed microsatellite markers. Genomic DNA (200 ng) was digested in NEB 4 buffer with the *Sbf1-HF* and *Nde1-HF* restriction enzymes (New England Biolabs) and ligated to adaptors all in a single unidirectional one-tube reaction [29]. The individual PCR-amplified libraries were pooled and size selected with the Pippin Prep instrument (Sage Science Inc.), using a 2% gel cassette (Cat No. CSD2010), and a size fractionation window of 340 to 420 bp was collected. Where required, the selected fragments were purified throughout the protocol with 1.1 volumes of AMPure XP (Beckman Coulter). Libraries were quantified on a Bioanalyzer High Sensitivity chip (Agilent Technologies), and a concentration of 35 pM selected for the library to be templated onto Ion Sphere Particles (ISPs). This was performed with an Ion Torrent OneTouch 2 (Thermofisher Scientific), and the Ion Torrent PGM Template OT2 400 Kit (Cat No. 4479878 with MAN0007218). Templating efficiency was monitored after enrichment using the Ion Sphere Quality Control kit (Cat. No. 4468656). The ISP libraries were then sequenced using the Ion Torrent PGM (Thermofisher Scientific), based on 316 v2 chips (Cat. No. 4488149). Fastq files were produced after base calling and de-replication with the Torrent Suite software version 4.0.2. The raw sequence reads generated from Ion Torrent sequencer were deposited in the Sequence Read Archive (SRA) database (accession number PRJNA595715). Sequence files were processed to identify microsatellite containing motifs and primer pairs which were designed based on both consensus and singleton sequences with the QDD program [30]. Consensus sequences were obtained with a default threshold of 95% identity being applied to merge reads with a high similarity.

### Validation of primers and microsatellite genotyping

Primer pairs were examined for efficiency with additional PCR reactions for both positive amplification and the ease of microsatellite identification in other species of *Heracleum* including *H*. *persicum*, *H*. *gorganicum and H*. *antasiaticum*, *H*. *anisactis*, *H*. *pastinasifolium* and *H*. *transcaucasicum* and *H*. *lasiopetalum* as listed in Table 1. Twenty five microsatellite markers were selected to test for amplification against a panel of 48 individuals from species of *Heracleum* (Table 1). Each PCR reaction contained 2.5 μl of Taq DNA Master Mix Red 2× (Amplicon, Denmark), 0.2 μM of each forward and reverse primer and 1 μl of genomic DNA (ca. 15 ng) in a final volume of 5 μl. Thermal cycles consisted of the initial denaturation at 95°C for 5 min, 30 cycles at 95 °C for 30s, 60 °C for 45s and 72 °C for 45s, followed by and 72 °C for 10 min in a Gene Amp 9700 thermal cycler (Applied Biosystems, USA). PCR products were checked for amplification using a 2% agarose gel.

To examine the efficiency of the developed markers in screening for genetic variation, the allele sizes for microsatellite loci were determined using a polyacrylamide gel. The procedure to prepare the polyacrylamide gel was identical to that already described in Movahedi et al. [31]. The size of the alleles were determined using a GeneRuler 50bp DNA ladder (Thermo Fisher Scientific).

### Data analysis

Clustering analysis was conducted based on pairwise genetic distances among populations, *Da* [32], using the Populations1.2.32 program. A two dimensional plot of principal coordinate analysis obtained based on genetic distance was constructed by plotting the two first components. In addition, population genetic structure was determined with a Bayesian approach, implemented as the Markov chain Monte Carlo (MCMC) algorithm, in Structure V.2.3.4 [33], to evaluate the likely population of origin of individuals. We used an admixture model with correlated allele frequency model and a 1.5 × 10^5^ burn-in period followed by 3 × 10^5^ iterations to compute the parameters. For each *K*, ranging from *K* = 1 to *K* = 6, 20 independent runs were applied. The likely optimum number of genetic clusters was determined by plotting the mean estimated ln probability of data, Pr(D), for each K and the plot was obtained with Structure Harvester [34]. For graphic visualization of the Structure results, we utilized the ClumpAK algorithm [35]. This enabled us to compare the results of independent runs and to estimate the similarity across Q matrices, and a plot was constructed to illustrate the proportions of memberships of individuals to each inferred cluster estimated from highly similar runs.

## Results

In total, 367 051 reads with average length of 229 bp were obtained from the three studied *H*. *rechingeri* individuals. The results revealed that 7886 reads (2.1% of total) contained microsatellite motifs with two, three, four, five and six nucleotide repeats, respectively. From these reads, 54 singleton and consensus sequences contained adequate flanking regions next to the microsatellite repeats enabling the design of primers (S1 Table). Twenty five loci were selected randomly to test for PCR amplification, and 19 primer pairs were successfully amplified in *H*. *rechingeri* and produced amplicons with the expected size. The nucleotide sequences of amplified loci were submitted to GenBank with accession number KX928682.1-KX928692.1 (Table 3).

**Table 3:** Specifications of microsatellite loci used to test for amplification. The annealing temperature for all primers was 60 °C.

Locus name	Primer sequences (5'-3')		No. reads	Motif	Amplicon size (bp)	GenBank accession No.
	Forward	Reverse				
Hrech436	CAGGACAGGAGAGGTCCAAG	CTAATTGTGAGGCTTGCGTG	1	(AT)_5_	83	KX928682.1
Hrech870	TCTTGCATATACATAATATCGCCC	GCTCTTCCGATCTCCTCAGC	1	(AT)_5_	325	KX928692.1
Hrech482	TGCTTTATATTATTGCTGGGCTT	CTCTACGTCTGTTGTGTTGTGA	1	(AT)_8_	83	KX928686.1
Hrech653	CATCCATGGTTTCGGCGG	GATTCGATTGCTTCAGGACG	1	(CG)_5_	92	
Hrech119	CCGCATTGGCATTAGAATTT	ATGGAGGAGGGATTTGATGA	1	(ATC)_5_	237	KX928684.1
Hrech1161	TGACTGTGATTGCTACTGTTTATCTC	TCCTGTCCTGTGAAGTGGTG	1	(AC)_5_	101	
Hrech2036	CGGGAATGTCCTGGTATCTG	GCTCTTCCGATCTCCTCAGC	1	(AT)_5_	319	KX928690.1
Hrech11_37	CATCTACGAAAGTGGTGGTCC	GCTCTTCCGATCTCCTCAGC	37	(TG)5	325	
Hrech1_9	GAAAGGCTGATCAAGACGGA	GCCTATGGGTCCTATGCTCC	9	(AT)12	198	
Hrech33_5	TTGTCTTGGCGAAGGATAGG	CGAGTAAGGTCTCGTGAGGG	5	(AC)5	299	KX928688.1
Hrech67_7	ATAGCTGTCCCTGGACCCTC	GCTCTTCCGATCTCCTCAGC	7	(AT)5	334	KX988700.1
Hrech1286	GGACTTGACAAACTAGGCCC	CGATCTGGACACCATCATTG	1	(AGG)5	236	KX928695.1
Hrech1247	AGGTCGAGTTCTTCTCCGGT	TTCCTCCACTTTCCCGAGAC	1	(AAG)5	277	KX928698.1
Hrech1728	GATTGCGGAAGGAGAAAGTG	GGGCCTTATCAGATGCCCTA	1	(AG)6	80	
Hrech2_4	CGGTTTGTGCCAGAGTTTGT	TCTCCTCAGCTACATCCCGT	4	(AG)7	299	KX928691.1
Hrech82	TGATTGTTTCCTCGTTTCAGAG	ATTCGGAAGATCAAAGCACG	1	(AT)5	183	KX928683.1
Hrech7_2	CAGCCGTAGTACAGAAGGCA	ATTCTGGCAGTGGAGGTCTG	2	(AG)6	256	KX928697.1
Hrech18_20	CGCACAATATGGACAAATCC	TCTTCACTCTCCTCTTTGTCCC	20	(AAG)20	177	KX928699.1
Hrech3_80	CAACAATAATCAAGTTCTGAGCA	TGCAGAGTGAAGTGTGTCTTCTT	80	(TC)5	128	KX928694.1
Hrech5_160	GACTCTGCACAATCTGGTGA	TATTCCTGTTGCCCTGCATT	160	(AC)5	279	KX928685.1
Hrech1250	TTTACAAGAATCGAGATGTGAA	CTCATGAGCAAATGATGGAAA	1	(AG)5	101	
Hrech1296	ATGTGTTCTTGTGTGAGAAAGGTG	TACGACACCACCAACTCAACC	1	(AG)5	180	KX928687.1
Hrech54_108	TGCTGAGCAGACCTTACTAACTT	TGAGGTAGTGGTTCCAAACTCAT	108	(AC)5	170	KX928696.1
Hrech60_5	TGACAGTCAAATCAGCCACA	GAGTGGTTTCTATTAGAGTAAGACCC	5	(AT)12	95	KX928693.1
Hrech45_53	GGTGCTACAGCGATAAGCAA	TGATTAGTGGACTCTATGTAGTGAGC	53	(TG)_9_	238	KX928689.1

**Fig 1:**
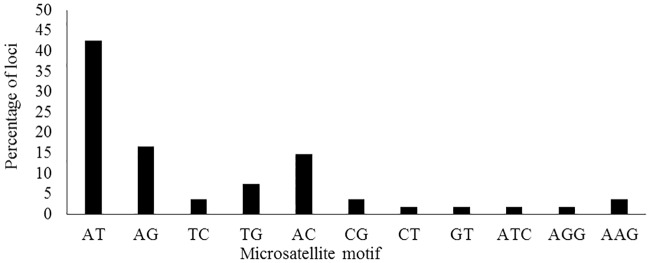
The frequency and nucleotide composition of microsatellite motifs identified across all reads obtained by ddRADseq.

Dinucleotide repeats (AT)_n_, (AG)_n_, and (AC)_n_ constituted 43%, 17%, and 15% of the microsatellite repeats, respectively (Fig 1, S1 Table). In addition, eight percent of the total repeats represented by the triplet nucleotides AAG (4%), AGG (2%) and ATC (2%).

Six loci comprised more than two alleles across all individuals from the studied species (Table 4). However *H*.*rech482* consisted of only two invariant alleles throughout all individuals and therefore this locus was excluded from all further analyses. Thus, 26% of loci were polymorphic across all individuals. A total of 17 alleles were observed across polymorphic loci, with an average 3.4 alleles per locus. The number of alleles varied between two (*H*.*rech3_80* and *H*.*rech436*) and six (*Hrech60_5*). All alleles were shared in at least two populations, thus no private allele was found across populations. Because of limited number of samples used in most populations the within-population statistics were not computed.

**Table 4:** The number of alleles and allele size range variation for amplified microsatellite loci which exhibited polymorphism amongst the 48 individuals of *Heracleum*.

Locus	Motif	Number of alleles	Allele size range (bp)
*H*.*rech60_5*	(AT)_n_	6	87–97
*H*.*rech60_5*	(AG)_n_	2	123–128
*H*.*rech1286*	(AGG)_n_	3	233–236
*H*.*rech1286*	(AT)_n_	2	83–85
*H*.*rech7_2*	(AG)_n_	4	256–261

Testing the cross-amplification of 19 microsatellite markers on 17 populations from eight different species of *Heracleum* showed that 100% and 94% of markers were successfully amplified in the thick-stem (*H*. *persicum*, *H*. *gorganicum* and *H*. *antasiaticum*) and thin-stem (*H*. *anisactis*, *H*. *pastinasifolium* and *H*. *transcaucasicum*) species, respectively. Also seven out of 19 loci (37%) were amplified in *H*. *lasiopetalum*.

**Fig 2:**
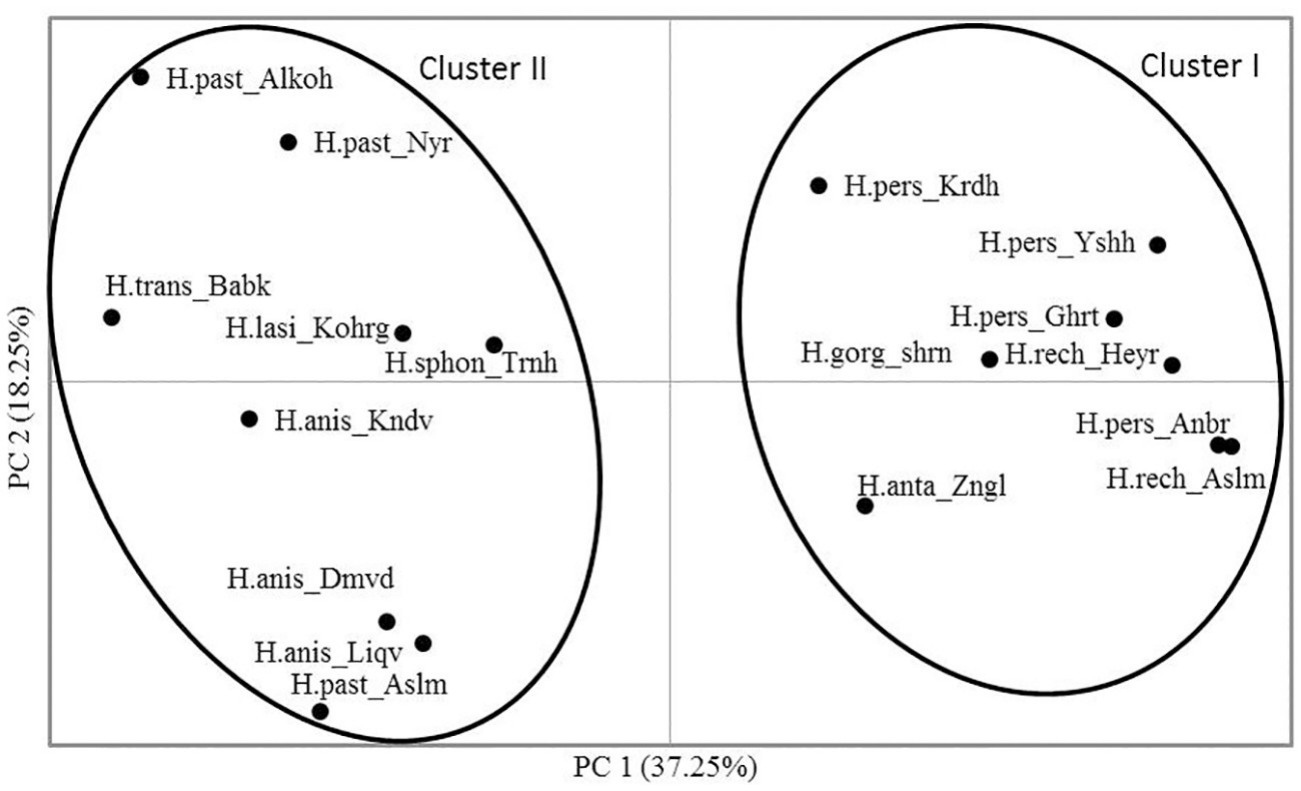
A two dimensional plot which captures 55% of the total variation obtained from microsatellite data exhibiting the variation within and among the species of *Heracleum* (*H*. *pastinasifolium*, *H*. *transcacasicum*, *H*. *persicum*, *H*. *rechingeri*, *H*. *gorganicum*, *H*. *lasiopetalum*, *H*. *antasiaticum* and *H*. *sphondylium* are abbreviated as H.past, H.trans, H.pers, H.rech, H.gorg, H. lasi, H.anta and H.sphon respectively; see Table 1 for details).

**Fig 3:**
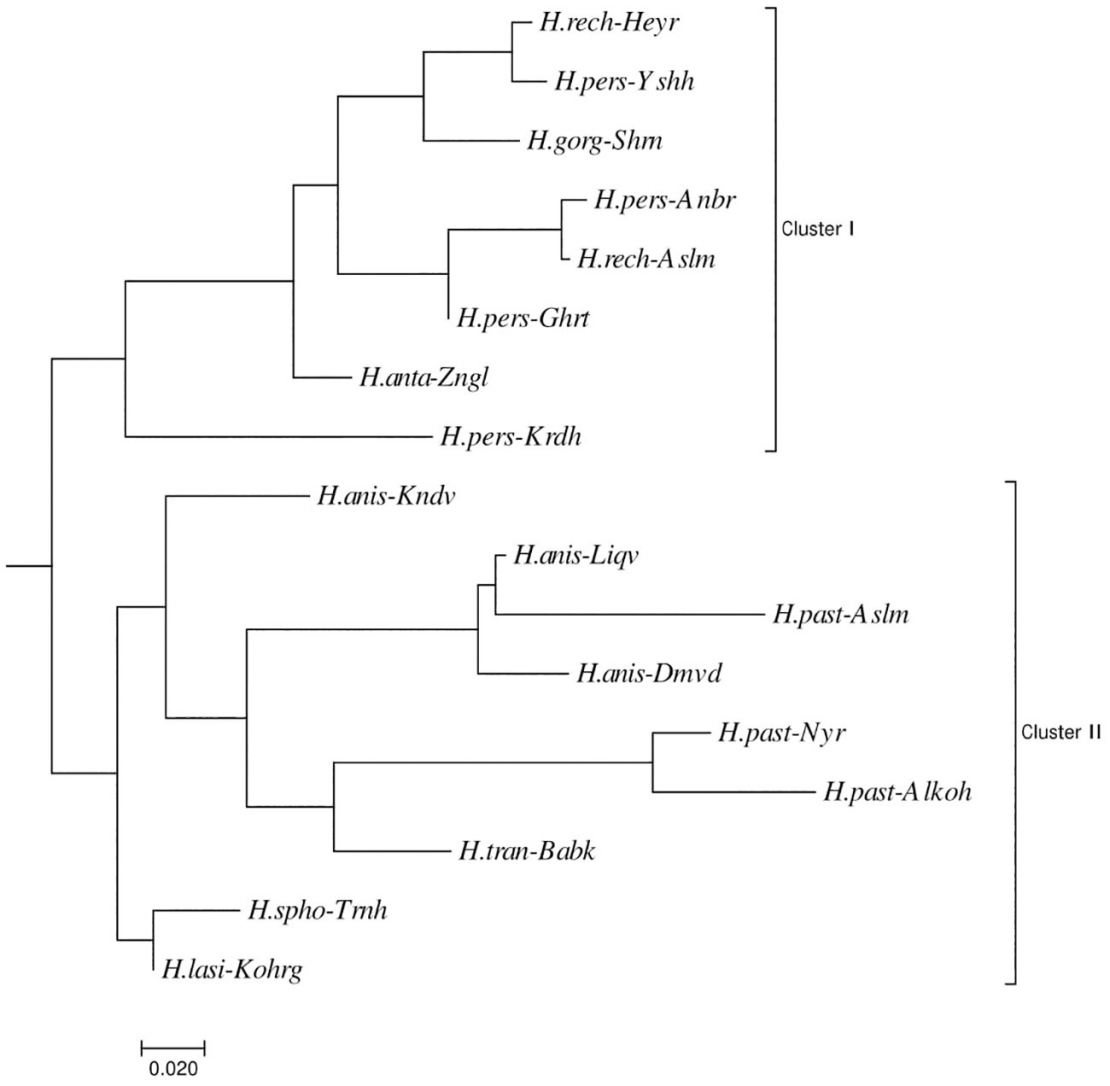
The clustering analysis using a neighbor joining approach obtained based on Nei et al., [32] Da genetic distance. The suffixes represent the abbreviation for the populations (see Fig 2 and Table 1 for details).

We analyzed the genetic structure of the *Heracleum* populations with both model-based Bayesian structure and by distance-based analyses. Principal coordinate and clustering analyses showed that the group of thick-stemmed species including *H*. *persicum*, *H*. *rechingeri*, *H*. *gorganicum* and *H*. *antasiaticum* were recovered together as a single cluster (Figs 2 and 3).

**Fig 4:**
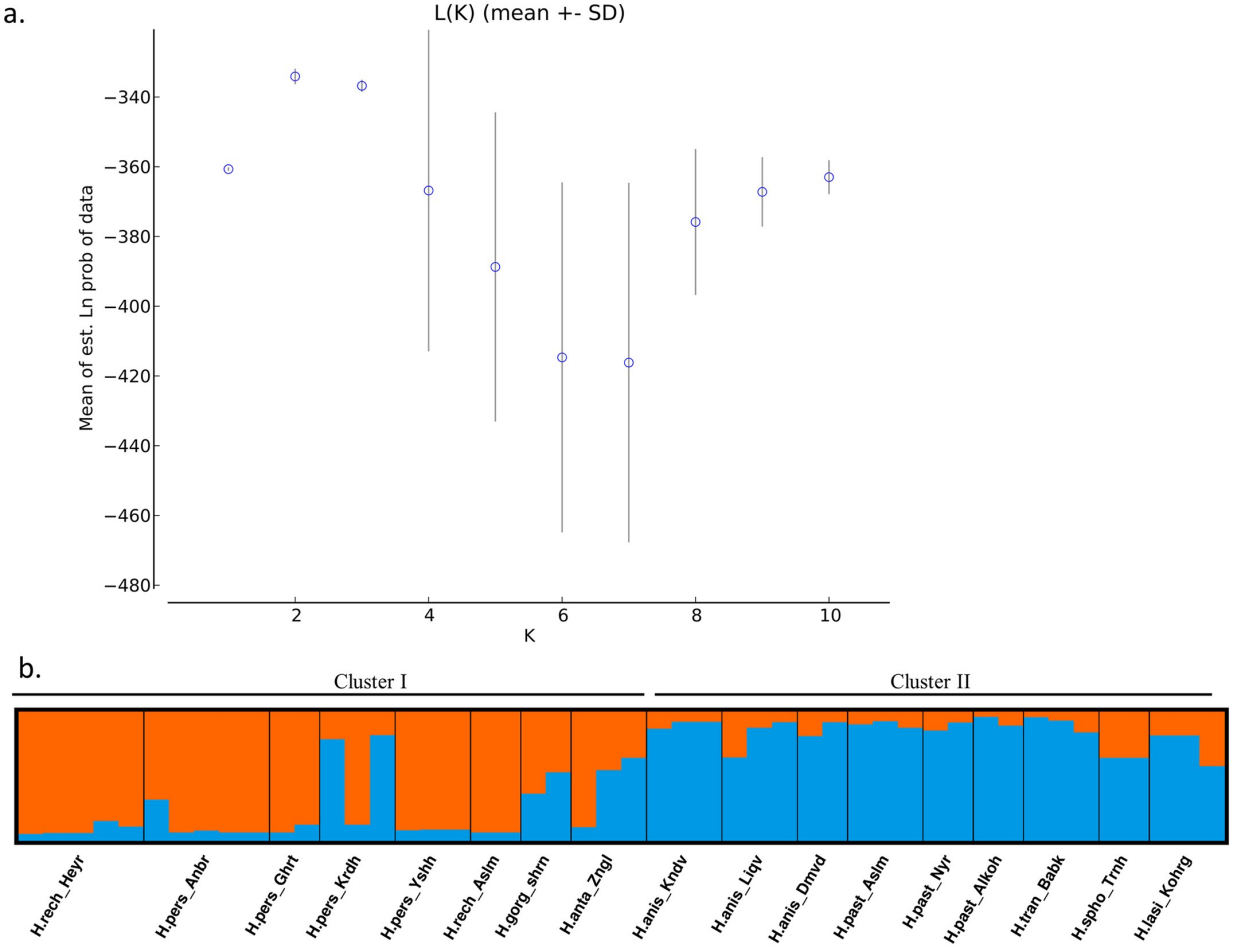
The results of Bayesian structure analysis in 48 individuals of *Heracleum* based on microsatellite data; a). The plot of mean estimated ln probability of data (±SD) obtained from 20 independent runs for each K. b). The proportion of memberships of individuals illustrating two main genetic clusters. Orange and blue colors mainly represent thick-stem and thin-stem species, respectively. Each individual is represented by a vertical bar.

The results of model-based clustering analysis suggested that the model with *K* = 2 was substantially better than other models (Fig 4a). A similar result to that observed by distance-based clustering was also obtained by model-based Bayesian genetic structure analyses. This exhibited two distinct genetic clusters, each corresponding to the thick-stemmed and thin- stemmed species groups, respectively (Fig 4b).

## Discussion

Novel microsatellite markers were obtained by screening ddRADseq next generation data from *H*. *rechingeri*. The development of these microsatellite markers then allowed the successful cross-amplification of those loci in congeneric taxa. We found that these markers proved to be efficient in distinguishing between the major sections of *Heracleum*, including the thick- and thin-stemmed species (Table 2). However, these markers were not able to distinguish and resolve sister taxa of the species complex mainly within the sections of Pubescentia and Wendia [14].

### Efficiency of ddRADseq in developing microsatellite markers

Previous investigations have reported (GA)_n_ as an abundant motif in various plant species (e.g. see [36] for a review). Our results, on the other hand, show that (AT)_n_ is a far more abundant dinucleotide repeat, but (AG)_n_ is also an abundant repeat in the taxa studied here. The percentages of total reads containing microsatellite motifs may vary among species and the types of sequencing methodology. A comparison amongst sequences obtained by traditional Sanger sequencing and NGS techniques, based on a review of 71 different studies, showed that NGS was approximately 40 times more effective in microsatellite marker development than Sanger sequencing [25]. In comparison, the development of microsatellite markers in *Satureja bachtiarica* and *Heracleum persicum* using 454 sequencing with an enrichment step for microsatellite loci, resulted in a discovery of 16% (8371 out of 51051 sequences) and 15% (3904 out of 25951) microsatellite motifs, respectively [2, 31]. RADseq data have also recently been used to identify microsatellites markers in various organisms, including several plant species. The application of RADseq data in *Solanum melongena* and *Cedrus atlantica*, has shown that 0.02% (2000 reads) and 8.5% (5714 reads) consisted of microsatellite motifs, respectively [37, 38]. Likewise, screening the next generation sequence data obtained by ddRADseq in *Eleusine coracana* and *Taxus florinii* have shown that 0.15% (10327 reads out of 6810971) and 1% (94851 of 8823053 reads) contain microsatellite motifs, respectively [39, 40]. The output of the present ddRADseq analyses, 2.1%, is comparable to that reported in other studies.

### Taxonomic application of microsatellite markers

Both model-based and distance-based clustering methods revealed two major genetic clusters, each representing a morphologically distinct group. Each genetic cluster consists of almost all samples from either thick-stemmed or thin-stemmed species, respectively. Our results also show that populations of *H*. *persicum* do not form a distinct group compared to the other closely related sister species, including *H*. *rechingeri* (Figs 2, 3 and 4b, Table 2). Based on taxonomic records there are few phenotypic traits that have been used as reliable taxonomic criteria to discriminate between *H*. *persicum* and *H*. *rechingeri* (Table 2). In Flora Iranica the presence of abaxial leaf trichome is listed as a diagnostic criteria in the latter species [14]. However, other molecular data from these species, including an ITS and *trnL-trnF* nucleotide analysis, together with a volatile oil composition study, also confirm our observed pattern which suggests a lack of differentiation amongst *H*. *persicum* and *H*. *rechingeri* populations [4, 6, 12], and perhaps that the abaxial leaf trichome is an unreliable trait in resolving *H*. *rechingeri* and *H*. *persicum*. Since inter-specific hybridization has been reported to be prevalent in *Heracleum* [2, 3], the observed patterns can also be interpreted as a lack of fixed differentiation resulting from an introgression of alleles between the congeneric species.

*H*. *antasiaticum* has several distinctive morphological traits including leaf and fruit shape and trichome density, which when taken together, distinguish it from the other thick-stemmed species (Table 2). Our clustering analyses confirm the genetic differentiation among this and other species. Three thin-stemmed species, *H*. *pastinacifolium*, *H*. *anisactis* and *H*. *transcacasicum* are also clearly distinct from the former group. In addition, the three populations from *H*. *pastinacifolium* are not genetically differentiated from populations of a morphologically similar species, *H*. *anisactis* and *H*. *transcacasicum*. Similar studies using ITS and *rpl32-trnL* nucleotide sequences, or from the analysis of the chemical constituents of essential oils derived from the fruits, have also delimited the thin- and thick stem species, even though *H*. *pastinacifolium* and *H*. *anisactis* remain similar [4, 12]. Limited numbers of cross-amplified microsatellite markers in *H*. *lasiopetalum* reveal a distant relatedness of this species within *Heracleum*. This species is morphologically different from the other study species and occurs in an alpine region of the Zagros mountains located in western part of Iran. This observation is also congruent with earlier results obtained from the few nuclear and chloroplast specific sequences that have been analysed to date [12].

Several taxonomic characters vary amongst *Heralceum* species, more specifically within the thick- and thin stemmed species complexes 14. However, there have been few reports of intraspecific variability in these traits. Our field observations indicate considerable variation between adult individuals within a given population. The observed morphological variability includes number of umbellate on the main umbel, which is described as the main taxonomic character that distinguishes *H*. *pastinacifolium* and *H*. *anisactis* [14]. This trait is highly variable within populations of *H*. *anisactis* and has overlapping trait variability values found in *H*. *pastinacifolium*. Interestingly, *H*. *anisactis* is listed as an endemic species in Iran with a narrower distribution range than that found for the *H*. *pastinacifolium* species. Our results suggest that *H*. *anisactis* and *H*. *pastinacifolium* are actually synonymous. Although the number of polymorphic microsatellite loci is limited in our study, the observed pattern is similar to that reported in earlier studies [4, 6, 12]. An additional systematic analysis by screening novel microsatellite markers, phenotypic and anatomical traits across several individuals for multiple populations within species might be required to depict the uniqueness of previously identified sister taxa.

## Conclusions

This study demonstrates the utility of microsatellite markers in studying complex species with otherwise weak taxonomic signals. The present analysis suggests that the thin-stemmed species (*H*. *anisactis* and *H*. *pastinasifolium*) and thick-stemmed species (*H*. *rechingeri* and *H*. *persicum*) are more closely related than what is perhaps expected from their described diagnostic taxonomic criteria. Long sequence reads obtained by ddRADseq provide a valuable tool to develop and deploy novel microsatellite markers, and provide a time and cost-effective approach for a genus that contains both endemic and invasive species.

## Supporting information

S1 Table Generic information of 54 microsatellite loci identified based on ddRADseq data in *Heracleum*. (XLSX)
